# Robot‑assisted resection of a rare bladder tumor facilitated by perioperative bladder wall tattooing

**DOI:** 10.20452/wiitm.2025.17932

**Published:** 2025-03-24

**Authors:** Michal Balík, Pavel Navráti, Lucie Šmejkalová, Miloš Broďák

**Affiliations:** Department of Urology, University Hospital Hradec Králové, Hradec Králové, Czech Republic; Department of Surgery, Charles University, Faculty of Medicine in Hradec Králové, Hradec Králové, Czech Republic; Department of Central Operating Theaters and Sterilization, University Hospital Hradec Králové, Hradec Králové, Czech Republic

**Keywords:** bladder wall tattooing, inflammatory myofibroblastic tumor, robot‑assisted urinary bladder resection

## Abstract

Inflammatory myofibroblastic tumors (IMTs) of the bladder are rare, with a limited number of cases reported in the literature. Complete resection with negative margins is essential to reduce the risk of recurrence, while bladder function preservation is also crucial for the patient. This study describes a 56‑year‑old patient with a bladder dome IMT managed using robot‑assisted partial cystectomy facili‑ tated by perioperative cystoscopic tattooing to precisely demarcate the tumor margins. The procedure began with cystoscopic tattooing of the lesion using Black Eye dye, followed by robotic resection with the da Vinci Xi system. Complete transmural resection and a 2‑layer bladder closure were performed, with preservation of the bladder capacity. The patient experienced minimal blood loss, no intraopera‑ tive complications, and was discharged 2 days after the procedure. Follow‑up examinations, including cystoscopy and computed tomography, confirmed no recurrence 12 months after surgery. Cystoscopic tattooing facilitated clear intraoperative tumor localization, enabling precise resection and minimal bladder wall loss. This approach addressed a key challenge of robotic bladder surgery—lack of tactile feedback—while maintaining functional outcomes. Robot‑assisted partial cystectomy with cystoscopic tattooing represents a promising alternative to maximal transurethral resection, especially in the context of bladder‑sparing trimodal therapy, for patients who are not eligible for or unwilling to undergo radical cystectomy. This technique is particularly relevant given the increasing focus on minimally invasive procedures and advancements in systemic therapy. In the future, this method could be adapted for ureteral robotic surgeries to enhance lesion localization.

## INTRODUCTION

Inflammatory myofibroblastic tumors (IMTs), sometimes also referred to as inflammatory pseudotumors or pseudosarcomatoid myofibroblastic proliferations, are rare. To date, only a few hundred cases have been reported in the literature, mostly in pediatric patients.^1^

Men are affected 3.2 times more often than women. One‑third of IMTs are found in the lungs; other typical locations include the mediastinum, retroperitoneum, and pelvis. IMTs can develop as a result of inflammation (most often in the lungs) or after surgery (eg, transurethral resection). However, spontaneous IMT occurrence is the most frequent.^2^ According to the World Health Organization, extrapulmonary IMTs are considered intermediate‑risk tumors due to a 25% incidence of local recurrence and an up to 5% risk of distant metastases. The risk of local recurrence is in‑ creased by multilocularity or a surgically inaccessible location (eg, ureteral orifice).^3^

Complete removal of the lesion with achievement of negative surgical margins is essential for patient prognosis. At the same time, preservation of the bladder function is key to prevent deterioration in patient quality of life.

Cystoscopic tattooing of bladder lesion margins with a dye was first described in the context of laparoscopic partial cystectomy in 2012.^4^ With the growing experience in robotic surgery and the expanding portfolio of procedures performed using robotic systems, we considered this method worth adapting to the specific requirements of robot‑assisted bladder resections.

**FIGURE 1:**
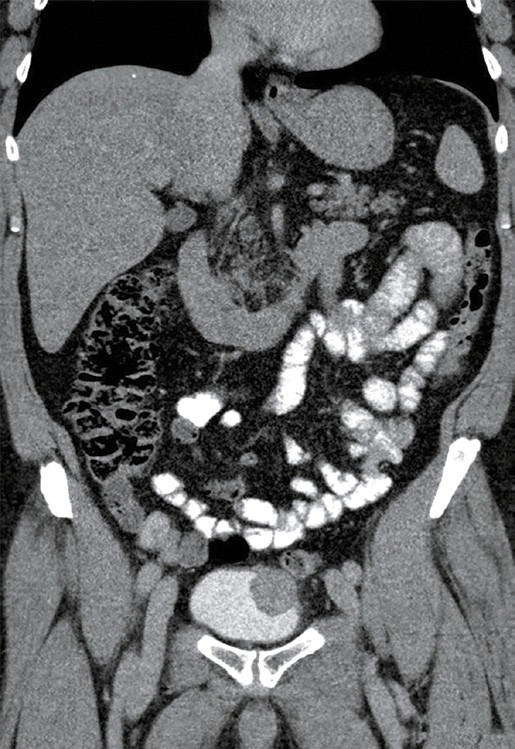
Computed tomography scan in the anteroposterior projection before surgery (tumor indicated by the arrow)

**FIGURE 2:**
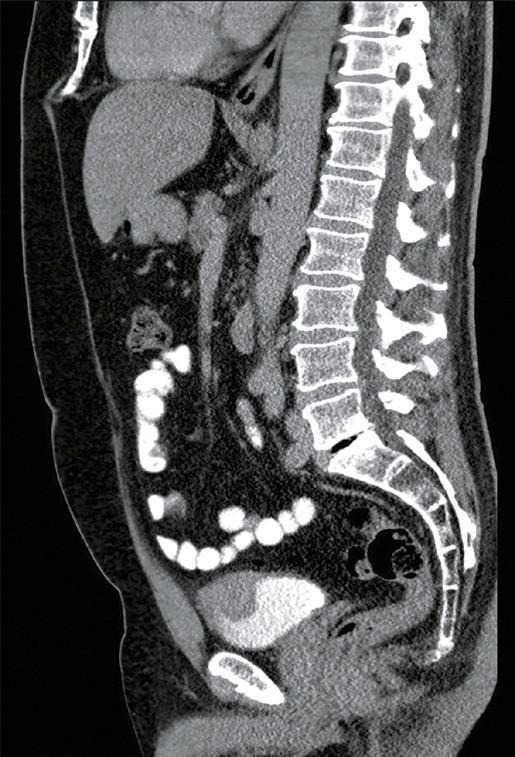
Computed tomography scan in the lateral projection before surgery (tumor indicated by the arrow)

**FIGURE 3:**
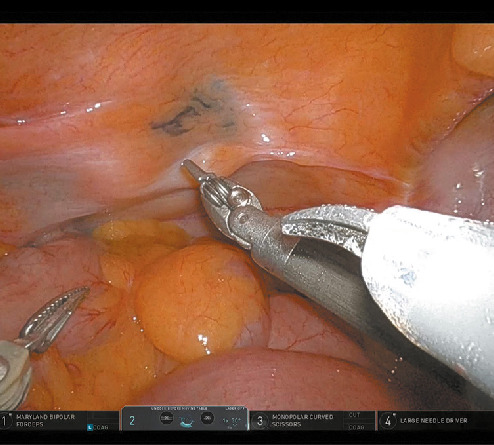
Intraoperative photograph taken during the robotic procedure showing the area in the bladder (parietal peritoneum) marked with a dye (arrow)

## MATERIALS AND METHODS

A 56‑year‑old patient with a history of recurrent macroscopic hematuria underwent cystoscopy, which showed a polypoid mass in the bladder dome. Based on computed tomography (CT) findings ( FIGURE 1 and FIGURE 2), we suspected that the mass invaded perivesical fat. A transurethral resection biopsy was performed; however, the procedure was not radical enough due to a risk of surrounding bowel loop injury. Histologic examination of the biopsy specimen confirmed IMT of the bladder, and the patient was scheduled for robot‑assisted bladder dome resection. Our aim was to make the procedure sufficiently radical (avoiding a positive surgical margin) to resect the entire tumor while preserving the bladder capacity. We decided to use lesion ink labeling to facilitate tumor localization, based on previous reports on the application of this method in abdominal laparoscopic procedures.^5^

The surgery started with cystoscopy, and several drops of Black Eye dye (The Standard Co., Ltd., Gunpo‑Si, Korea) were injected submucosally, approximately 1 cm from the tumor, using a Williams needle (45 cm/5 F; Cook Inc., Bloomington, Indiana, United States). Subsequently, capnoperitoneum was achieved using a Veress needle. Four robotic ports were introduced at the level of the umbilicus, similarly to the protocol for robotic prostatectomy, and the da Vinci Xi surgical system (Intuitive Surgical, Sunnyvale, California, United States) was docked. The placement of the ports was as follows: arm 1, Maryland bi‑ polar forceps; arm 2, camera; arm 3, monopolar curved scissors; arm 4, a large needle driver, with the assistant port placed at the left medioclavicular line below the umbilicus.

The parietal peritoneum over the bladder dome showed no evidence of a tumor. The dyed spot, on the other hand, was clearly visible and allowed for safe entry into the bladder next to the lesion (FIGURE 3). After complete transmural resection (including the parietal peritoneum), bladder closure was performed with 2 layers of continued Vloc 90 absorbable suture (Medtronic, New Haven, Connecticut, United States). After checking the tightness of the suture by filling the bladder with 200 of ml saline solution, a drain was introduced through the lateral port into the Douglas pouch. The specimen was placed in a bag and extracted through the umbilical wound

## RESULTS

The total procedure time, including cystoscopy, was 60 minutes. No instrument ex‑ change was needed. Blood loss was negligible.

**FIGURE 4:**
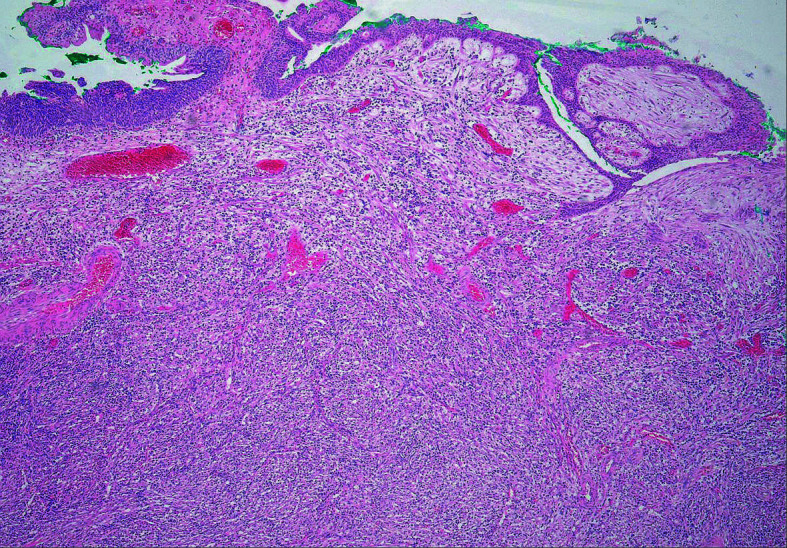
Histological examination of the excised specimen showing inflammatory myofibroblastic proliferation (hematoxylin‑eosin staining, magnification×100)

**FIGURE 5:**
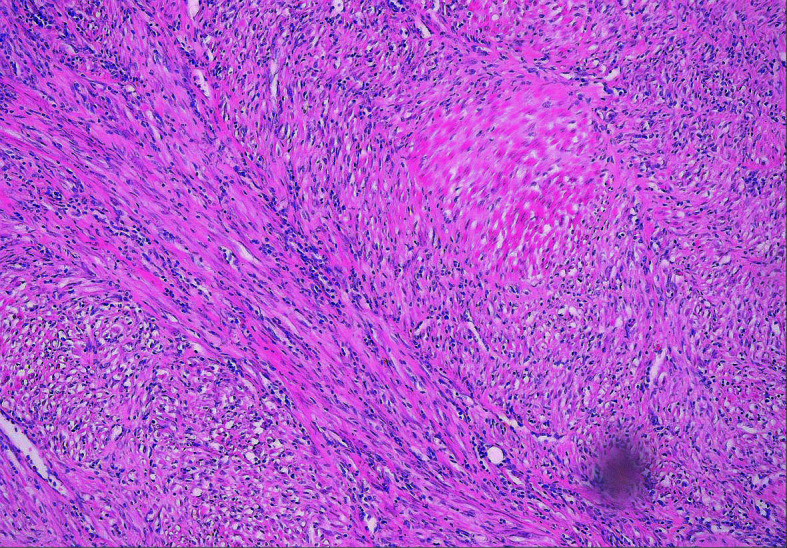
Histological examination of the excised specimen showing inflammatory myofibroblastic proliferation (hematoxylin‑eosin staining, magnification×400)

**FIGURE 6:**
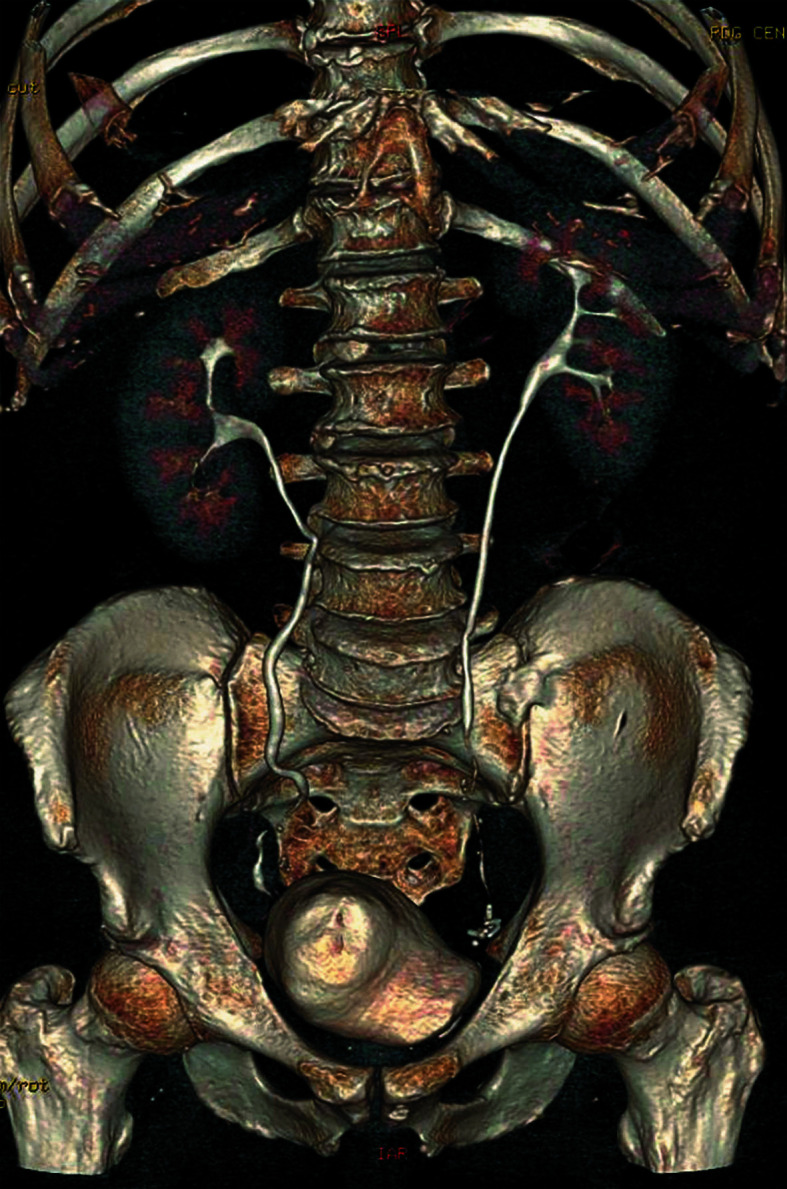
Three‑dimensional reconstruction of a computed tomography scan in the anteroposterior projection 6 months after surgery showing no signs of the disease recurrence

There were no periprocedural or anesthesia‑related complications.

After the procedure, the patient was kept under observation for 3 hours in the intermediary care unit, and then was transferred back to the standard care ward. On the following day, after mobilization, the drain was extracted from the peritoneal cavity. The patient was discharged a day later, with a permanent catheter left in place.

Follow‑up cystography was performed on post‑operative day 7. There was no contrast material leak, and the urinary catheter was removed. Histological examination confirmed IMT without a positive margin (FIGURE 4 and FIGURE 5). The evaluation was not affected by tattooing, as the dye did not reach the surgical margin of the specimen.

Follow‑up cystoscopy performed 3, 6, and 12 months after surgery showed a healed scar in the bladder dome, preserved bladder capacity, no recurrence of IMT, and a receding amount of the dye in the bladder wall. A follow‑up CT scan 6 months after surgery showed no signs of the disease recurrence FIGURE 6.

## DISCUSSION

In recent years, there has been a general tendency toward minimally invasive surgical procedures. This approach offers many benefits, such as minimal blood loss or tissue damage and faster recovery.6 Early mobilization seems to be the main protective factor against thromboembolic events.^7^^,^^8^

Precise localization of the lesion in the bladder fundamentally determines patient prognosis. Opening the bladder at the site of the lesion increases the risk of incomplete resection or im‑ plantation metastases. On the other hand, greater extent of bladder tissue excision increases the risk of compromising functional outcomes—impaired urinary bladder capacity and occurrence of irritative lower urinary tract symptoms.

Intraoperative localization of a bladder tumor during robot‑assisted laparoscopic surgery is challenging due to a lack of tactile feedback. An ultrasound probe could theoretically be used for this purpose; however, in contrast to renal tumors, bladder lesions tend to be rather flat. There‑ fore, identification of the tumor margins on ultrasound may not be sufficiently accurate. Tattooing seems a feasible, inexpensive, and safe localization method.

The gold standard treatment for muscle‑invasive bladder cancer (MIBC) is radical cystectomy. However, the 5‑year overall survival following this surgery is approximately 50%.9 Untreated advanced MIBC is often associated with un‑ controllable bleeding, which can result in painful bladder tamponades. Beyond its curative intent, radical (as well as partial) cystectomy has the potential to prevent such tamponades. Despite its benefits, radical cystectomy is associated with significant morbidity and mortality, largely due to complications arising from urinary diversion, a procedure involving reconstruction of the urinary tract using bowel segments. As a result, clinicians have explored alternative treatments for patients who are ineligible or unwilling to undergo radical cystectomy. Robot‑assisted partial cystectomy with perioperative cystoscopic tattooing of the bladder wall presents a promising alternative to maximal transurethral resection in the con‑ text of bladder‑sparing approaches, which have gained popularity as part of the trimodal therapy for MIBC, which combines surgery, radiation therapy, and chemotherapy. This approach is particularly relevant in light of the emerging possibilities in systemic therapy. Appropriate patient selection is crucial for achieving sufficient disease control with bladder‑sparing therapy. Optimal selection criteria include good bladder function and capacity, no history of pelvic radiotherapy, absence of carcinoma in situ, multilocularity, involvement of more than 30% of the bladder surface or trigonum, tumor stage below cT4, absence of hydronephrosis, tumor within a diverticulum, and a short life expectancy.^10^^,^^11^

To our best knowledge, this is the second re‑ port of bladder wall cystoscopic tattooing during laparoscopic partial cystectomy.^4^

## CONCLUSIONS

Robot‑assisted surgery combines the advantages of a minimally invasive approach with precision and dexterity comparable to those of open pelvic surgery.

We showed that tattooing of the bladder wall to facilitate precise resection of a benign but large tumor is a feasible approach. It allowed for sufficiently radical tumor removal and, at the same time, preservation of the bladder capacity.

In the future, the potential application of cystoscopic tattooing may be expanded to ureteral robot‑assisted surgeries to help specify the location of a ureteral lesion, such as a stricture or tumor. Partial cystectomy could be considered a part of trimodal therapy for bladder cancer for selected patients who are not eligible for or refuse to undergo radical cystectomy.

## AI Statement

This article has undergone language proofreading with This article has undergone language proofreading with the assistance of an artificial intelligence tool, specifically ChatGPT‑4 by OpenAI. The AI was used exclusively for enhancing the grammar, style, and clarity of the text. While the AI contributed to the content, the final text was reviewed, edited, and approved by the author to ensure accuracy, coherence, and originality. The author takes full responsibility for the content presented in this article
